# Insulin growth factor 1 like receptor (IGF-1R)

**DOI:** 10.1186/s12885-016-2796-x

**Published:** 2016-10-06

**Authors:** Gopal Iyer, James Price, Shay Bourgeois, Eric Armstrong, Shyhmin Huang, Paul M. Harari

**Affiliations:** Department of Human Oncology and the University of Wisconsin School of Medicine and Public Health, University of Wisconsin-Madison, Madison, 53792 USA

**Keywords:** Head and neck cancer, Clinical antibody cetuximab, Heterodimerization, Phosphorylation, Picropodophyllin

## Abstract

**Background:**

The epidermal growth factor receptor (EGFR) is frequently overexpressed in head and neck squamous cell carcinoma (HNSCC) and several other human cancers. Monoclonal antibodies, such as cetuximab that block EGFR signaling, have emerged as valuable molecular targeting agents in clinical cancer therapy. Prolonged exposure to cetuximab can result in cells acquiring resistance by a process that remains incompletely understood.

**Methods:**

In this study, we analyzed the immediate early molecular response of cetuximab on physical interactions between EGFR and Insulin growth factor 1 like receptor (IGF-1R) in head and neck cancer cells that are resistant to cetuximab. Co-immunoprecipitation, small molecule inhibitors against phospho-Src and IGF-1R, quantitative western blot of EGFR and Src phosphorylation, cell proliferation assays were used to suggest the role of IGF-1R mediated phosphorylation of specific tyrosine Y845 on EGFR via increased heterodimerization of EGFR and IGF-1R in cetuximab resistant cells.

**Results:**

Heterodimerization of EGFR with IGF-1R was increased in cetuximab resistant HNSCC cell line UMSCC6. Basal levels of phosphorylated EGFR Y845 showed significant increase in the presence of cetuximab. Surprisingly, this activated Y845 level was not inhibited in the presence of Src inhibitor PP1. Instead, inhibition of IGF-1R by picropodophyllin (PPP) reduced the EGFR Y845 levels. Taken together, these results suggest that heterodimerization of EGFR with IGF-1R can lead to increased activity of EGFR and may be an important platform for cetuximab mediated signaling in head and neck tumors that have become resistant to anti-EGFR therapy.

**Conclusions:**

EGFR-IGF-1R interaction has a functional consequence of phosphorylation of EGFR Y845 in cetuximab resistant HNSCC cells and dual targeting of EGFR and IGF-1R is a promising therapeutic strategy.

**Electronic supplementary material:**

The online version of this article (doi:10.1186/s12885-016-2796-x) contains supplementary material, which is available to authorized users.

## Background

Epidermal growth factor receptor (EGFR) is a receptor kinase that plays essential roles in development. The EGFR is overexpressed and mutated in several human cancers including the majority of cases of HNSCC [1]. 90 % of HNSCC patients have increased EGFR protein levels despite the lack of amplification of the EGFR locus [2]. In addition, the cancer genome atlas (TCGA) has identified amplification and mutation of EGFR in a proportion of human papillomavirus (HPV) positive and negative head and neck cancers [3]. This overexpression of EGFR leads to dysregulated signaling in HNSCC [4]. Inhibition of EGFR using either monoclonal antibodies (mAbs) against the extracellular domain or small molecule G-protein coupled receptors (TKI) inhibitors against the intracellular domain [5], combining mAbs with radiotherapy [6] and chemotherapy [7], have resulted in therapeutic benefits [8] including improvement in tumor response and overall survival in cancers [9–12]. For instance, the clinical anti-EGFR mAb cetuximab is capable of interfering with the ligand binding site of EGFR to downregulate downstream signaling pathways associated with cell proliferation. However, there is increasing evidence of acquired resistance to this antibody [13] necessitating alternate molecular targets and better strategies for effective treatment. The onset of EGFR resistance can trigger alternative signaling pathways through association with other receptor tyrosine kinases [14, 15] or G-protein coupled receptors (GPCRs) [16] to maintain the tumor phenotype but these precise mechanisms remain only partially understood.

Current technological improvements in genomic and proteomic platforms [17] have identified many promising targets for which inhibitors are being pursued. One such molecular target is Insulin-like growth factor receptor 1 (IGF-1R). Like EGFR, IGF-1R also plays a role in the maintenance of the oncogenic phenotype in various cancers [18] and is known to mediate anti-apoptotic signals and cell proliferation [19]. Interaction of insulin like growth factor I and II (IGF1 and IGF2) with IGF-1R is required for cell growth, proliferation and apoptosis [20] while IGF2- IGF1R interaction is not required for adult growth and development [21]. The recently reported head and neck cancer TCGA has identified 4 % amplification and mutation of IGF1R gene in HPV negative HNSCC patients [3]. Furthermore, activation of IGF-1R has been reported to induce resistance to EGFR TKIs [22].

In this study we investigated the response of HNSCC cell lines to cetuximab. We found that in cetuximab-resistant cells there is an increased heterodimerization of EGFR and IGF1R, in response to cetuximab. Furthermore, the inhibition of EGFR by the IGF-R inhibitor picropodophyllin (PPP) reduces the EGFR tyrosine 845 phosphorylation suggesting that the interaction has a consequence on downstream signaling pathways. These findings suggest that an early molecular event following cetuximab binding to EGFR leads to heterodimerization with IGF-1R to maintain survival and proliferation of resistant cells and suggests a potential mechanism for acquisition of resistance.

## Methods

All biochemical experiments, cell proliferation and RNA experiments were performed in triplicates or more.

### Cell lines

The human head and neck SCC6 (UM-SCC6) cells were kindly provided by Dr. Thomas E. Carey (University of Michigan, Ann Arbor, MI). SCC6 (parental) and cetuximab resistant clone (resistant) were cultured in Dulbecco’s Modified Eagle’s Media supplemented with 10 % FBS and 1 mg/mL hydrocortisone with 1× penicillin-streptomycin. Cetuximab resistant clones against EGFR were developed in SCC6 (parental) background as described previously [23]. All cell lines were tested for authenticity in accordance with ATCC guidelines.

### Reagents and antibodies

Cetuximab was provided by Imclone systems. Antibodies against total EGFR, IGF1R, Src, phospho-EGFR tyrosine 845, phospho-Src tyrosine 416, GAPDH and mouse immunoprecipitation (IP) specific antibody were obtained from Cell Signaling Technology (Beverly, MA). Anti-IGF1R for IP experiments was obtained from Life Technologies, (Carlsbad, CA). All other secondary antibodies were obtained from Cell Signaling Technology (Beverly, MA). 1-(1,1-dimethylethyl)-3-(4-methylphenyl)-1H-pyrazolo[3,4-d]pyrimidin-4-amine (PP1) Src inhibitor was obtained from **Biomol GmbH**. Picropodophyllin (PPP) and AG1024 was obtained from Santa Cruz Biotechnology Inc (Santa Cruz, CA). Dasatinib was obtained from Cayman chemicals (Ann Arbor, MI). Cell culture media and supplements were obtained from Life Technologies, Inc. (Carlsbad, CA).

### Protein extraction

SCC-6 sensitive and cetuximab resistant cells were extracted in a protein extraction buffer which consisted of 25 mM HEPES buffer, pH 7.9, 1 U/μLof Benzonase 1 mM MgCl, 5 mM EDTA and 1 tablet of EDTA free protease inhibitor and PhosSTOP (Roche) per 10 ml of buffer. To this, an optimized mixture of detergents was added comprised of 0.5 % TrX100, 0.5 % NP-40S, 0.5 % cholic acid sodium salt (Cholate), 0.25 % n-dodecyl-β-D-maltoside and 0.25 % 3-[(3 cholamidopropyl) dimethylammonio]-1-propanesulfonate (CHAPS). Cells were treated with 10 μg/ml cetuximab and harvested at 80 % confluence, washed two times with ice-cold PBS, scraped with cell scraper and pelleted at 20,000 g for 10 min and homogenized with motorized homogenizer for 1 min. The mixture was centrifuged at 20,000 g for 10 min at 4 °C. The resultant pellet was resuspended, rehomogenized and then recentrifuged at 20 000 g for 10 min at 4 °C. The supernatant was transferred into fresh tube and concentration was determined with the bicinchoninic acid (BCA) Protein Assay Reagent (Thermo Scientific).

### SDS-PAGE and western immunoblot analysis

**Fig. 1:**
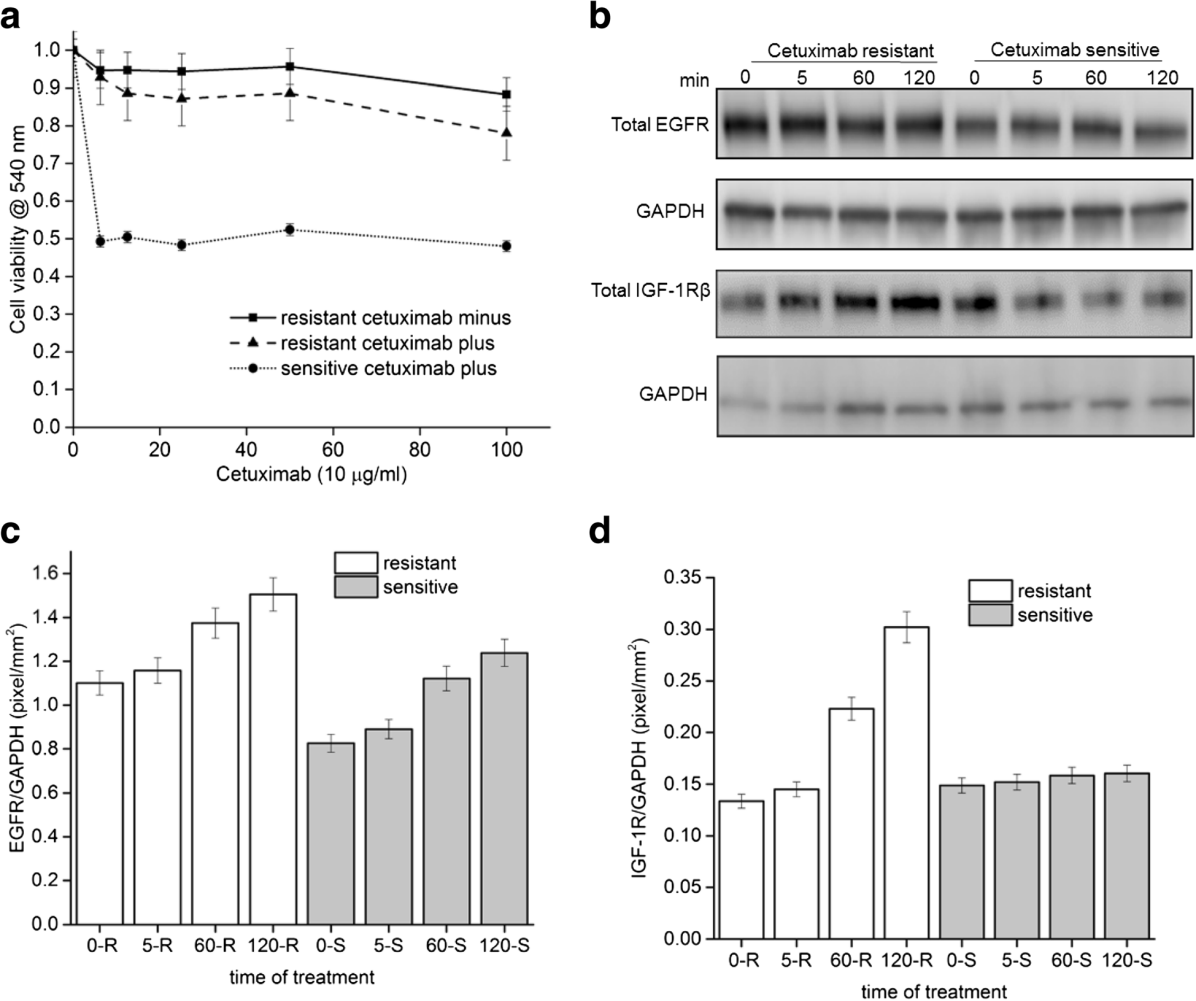
IGF-1R increase in resistant cells upon exposure to anti-EGFR antibody cetuximab. **a** UMSCC6 cetuximab resistant and sensitive cell lines at passage 17 were exposed to increasing concentration of cetuximab to measure cell viability by absorbance in a crystal violet assay (■) Resistant cells in the absence of cetuximab, (●) sensitive cells in the presence of cetuximab (▲) resistant cells in the presence of cetuximab. **b** Representative blot of total EGFR and IGF-1R show increased levels in cetuximab resistant cells relative to parental cells. **c**, **d** Quantitation of total EGFR and IGF-1R levels normalized to GAPDH from four independent experiments

Protein extracts was mixed with 4× Laemmli buffer Laemmli buffer (Bio-rad, Hercules, CA) boiled in a heating block at 95 °C for 5 min,loaded and electrophoretically separated onto a 4–20 % gradient tris-glycine polyacrylamide gel (Bio-rad) Subsequently, the gels were transferred to low fluorescent nitrocellulose membrane using the turbo-blot system (Bio-rad) following the manufacturer’s instructions. The membranes were stained with 0.1 % Ponceaus S in 1 % acetic acid for monitoring transfer and equal loading and blocked with 5 % nonfat dry milk. Following blocking, the membranes wereincubated with primary antibodies at 1:1000 dilution in 5 % BSA in Tris buffered saline with 0.1 % Tween-20 (TBST) at 4 °C overnight. Incubation with peroxidase-labeled secondary mouse or rabbit antibodies was carried out at was conducted at room tem-perature for 1 h, followed by 3 washes times 5 min with TBST. Bands were exposed after addition of the GE ECL Prime kit (GE healthcare, USA) and digitally recorded using a LI-COR Fc imaging system. All western blots were repeated in triplicate. For Figs. 1c, d and 4b, d, Immunoblots were imaged using an Odyssey infrared imaging system Fc (LI-COR Biosciences). Scan resolution of the instrument was fixed at 125 μm and images were acquired at integration times of 30 s. Quantification was performed with Image studio lite software. The median pixel intensity quantified for each band was normalized to GAPDH which served as the loading control. The experimental intensity values of experimental was divided with GAPDH and graphed with Originlab software. All experiments were performed in triplicates.

### Co-immunoprecipitation

UMSCC6 cetuximab sensitive and resistant cells were plated (on day 1) at a density of 4 million cells per 15 cm dish and treated with 10 μg/ml on the 3^rd^ day and harvested at 5, 60 and 120 min with 0 min serving as control untreated sample. Treated cells were harvested on day 3 by scraping cells with a cell scraper (Corning Inc., Corning, NY), and cells transferred to a 1.5 mL conical tube (Wilmad Glass, NJ) for centrifugation and lysis as described for immunoblot procedure above. 1 mg/ml of total protein was precleared with 30 ul of Protein G Dynabeads and incubated with preformed complexes of protein G Dynabeads (Invitrogen, Carlsbad, CA) and mouse anti- EGFR and rabbit IGF-1R antibodies (CST, Beverly, MA and Life Technologies, Carlsbad, CA) at room temperature was end to end mixed on a rocker for 30 min and further at 4 °C for 3 h. The antigen-antibody complexes were collected using a magnet and washed with 1× PBS containing 0.1 M NaCl, 0.5 M NaCl and 0 M salt progressively. Complexes were dissociated by incubating in reducing buffer (4× Laemmli buffer for 5 min at 95 °C. The boiled samples was cooled on ice and removed from beads using a magnet and loaded onto a 4–20 % gradient SDS-PAGE gel for western analyses.

### Quantification of mRNA expression

Relative mRNA levels of EGFR and IGF-1R were quantified via real-time PCR (RT-qPCR) using a Bio-Rad C1000 qPCR Detection System and Power SYBR Green PCR Master Mix as recommended by the manufacturer (Life Technologies). All reactions were performed in triplicate from RNA isolated from three independent biological experiments. The geometric mean of four housekeeping genes (β-Actin, β-Microglobulin, GAPDH, HPRT1) was calculated and used for normalization (Additional file 1: Table S1). Fold increases or decreases in gene expression after treatments were normalized to an untreated control sample at time zero.

### Cell viability assay

For Fig. 1a, cetuximab resistant and sensitive cells previously described in (23) were continuously exposed to increasing concentrations of cetuximab for over a period of 3 weeks to evaluate its sensitivity to cetuximab. Commencing with the IC_50_ of cetuximab, the exposure dose was progressively doubled every 10–14 days until 7 ~ 8 dose doublings had been successfully achieved. In parallel, controlled parental cells were exposed to corresponding vehicle, PBS. Exponentially growing cells were seeded in 6 well plates. Following the treatment, cells were then washed with PBS and fixed/stained with 0.5 % crystal violet. Plates were air dried overnight and dye was eluted with 0.1 M sodium citrate (pH 4.2) in ethanol (1:1). Elution was transferred to 96 well plates, and the absorbance was read at 540 nm to determine cell viability and untreated cells were normalized to 1.

### Cell proliferation assay

The CCK-8 assay was used to measure cytotoxicity, based on the conversion of a water-soluble tetrazolium salt, 2-(2-methoxy-4-nitrophenyl)-3-(4-nitrophenyl)-5-(2,4-disulfophenyl)-2H-tetrazolium, monosodium salt (WST-8), to a water-soluble formazan dye upon reduction by dehydrogenases in the presence of an electron carrier. HCECs (2.5 × 10^4^ cells/ml) were grown in 96-well plates for 48 h and treated with inhibitors PP1, Dasatinib, PPP with or without cetuximab antibody, wherever applicable. After 36 h, cells were washed, and the extent of cell growth was assessed using a CCK-8 assay (Dojindo, Kumamoto, Japan). CCK-8 solution (10 μl) was added to each well, followed by incubation for 2 h at 37 °C. The absorbance at 450 nm was determined by a multiplate reader (Lambda Bio-20; Beckman). Cell viability was expressed as a percentage of that of the control (untreated) cells. The mean values of the mean absorbance rates from eight wells and percentage coefficient of variation were calculated and graphed using Originlab software.

## Results

We sought to examine signaling activation in the context of acquired resistance to the anti-EGFR antibody, cetuximab, in the previously described squamous head and neck carcinoma line UMSCC6 (University of Michigan squamous cell carcinoma) [23]. We first confirmed the stability of the UMSCC6 cetuximab resistant cell lines to challenge with varying concentrations of cetuximab. For this purpose, resistant cells were maintained in media in the presence or absence of cetuximab for 17 passages and compared to parental cetuximab sensitive cells. Using a crystal violet assay, we established that the proliferative capacity of resistant cells remained unchanged irrespective of cetuximab in the media while the sensitive cells had 50 % proliferation at similar concentrations of cetuximab (Fig. 1a). Having established the resistance phenotype and observed 50 % reduction in proliferation in the sensitive cells, we performed all experiments at 10 μg/ml cetuximab concentration.

To determine the immediate early effects of perturbation on EGFR with cetuximab, we compared the levels of EGFR protein in both sensitive and resistant cell lines. Total EGFR levels increased modestly in both resistant and sensitive with increasing time of exposure to cetuximab (Fig. 1b and c). We next asked if cetuximab had any local effect on IGF-1R. IGF-1R, a RTK known to be involved in malignant transformation and expressed in tumors was upregulated in resistant cells when compared to sensitive cells (Fig. 1b and c). Furthermore, quantification of the immunoblots revealed that IGF-1R protein level was induced by two- fold within 2 h of exposure to cetuximab in resistant cells (Fig. 1c). In the same time period, EGFR levels are induced 1.4 and 1.2 fold respectively in resistant and sensitive cells (Fig. 1c). Taken together, the quantitative analyses suggested that cetuximab increased the levels of IGF-1R in resistant cells when compared to sensitive cells.

**Fig. 2:**
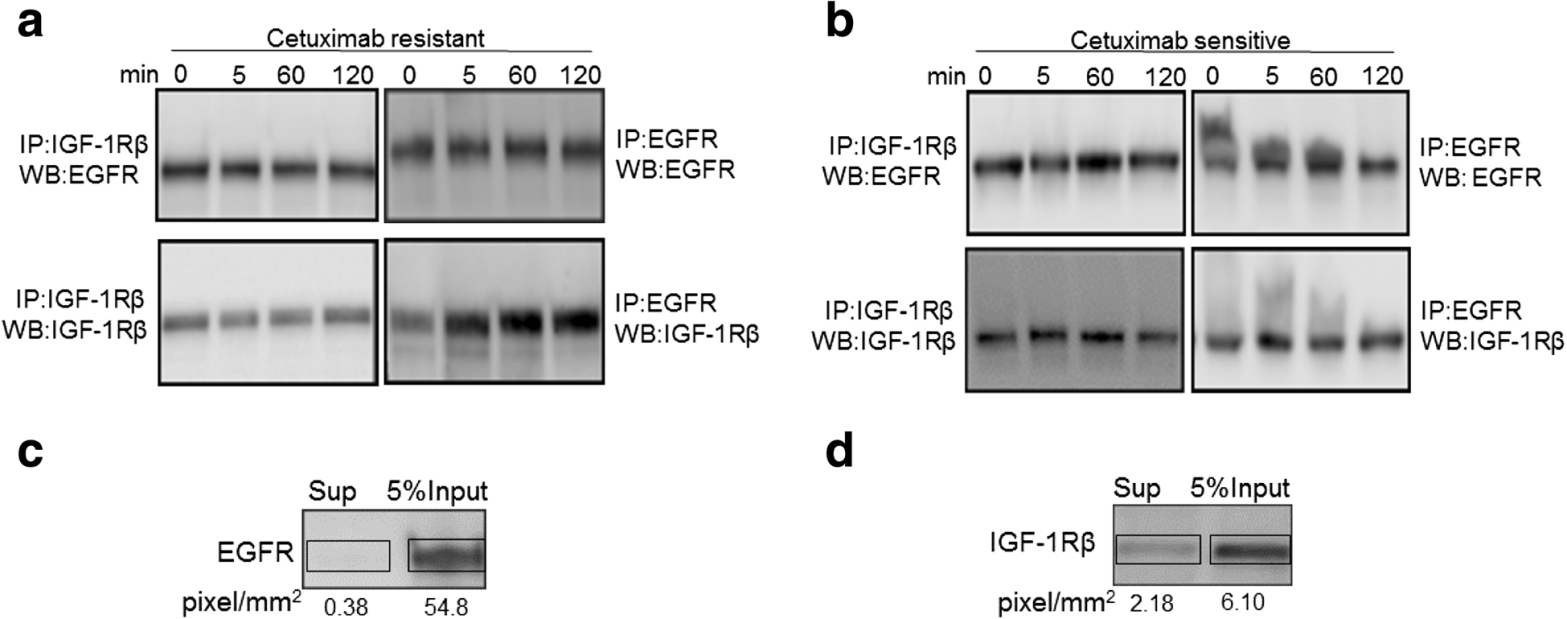
IGF-1R interaction with EGFR increases with cetuximab treatment in resistant cells. **a** Co-immunoprecipitation (IP) of EGFR and IGF-1Rβ in resistant cell lysates with increasing time of exposure to cetuximab. Reciprocal immunopreciptations with either EGFR or IGF1R are presented in right and left panels respectively. **b** As in A, but for sensitive cell lysates. **c** EGFR western blot in input and supernatant (sup) after IP with anti-EGFR in resistant cell lysate. 5 % of each sample is loaded. **d** as in C but for anti-IGF1R in resistant cell lysate

We next investigated whether this increased IGF1R level had a functional consequence in the SCC cells. Mechanistically, blocking the ligand binding site of EGF with cetuximab could result in signaling or activation of IGF-1R or sustained engagement of adaptor proteins via receptor heterodimerization, thus enabling an alternative pathway for survival and proliferation. Therefore we assessed the levels of heterodimerization of IGF-1R and EGFR in resistant and sensitive cells. In resistant cells treated with cetuximab, increasing amounts of IGF-1R protein were observed in the EGFR immunoprecipitate with time of exposure (Fig. 2a). The interaction of EGFR and IGF-1R was detected as early as 5 min and remained sustained to 120 min (Fig. 2a) mirroring the increasing levels of IGF1R total protein (Fig. 1c). By comparison, in sensitive cells, the relative amount of IGF1R co-immunoprecipitated with EGFR remained constant with time (Fig. 2b). In both cell lines, the reciprocal IGF-1R co-immunoprecipitation did not reveal increasing amounts of EGFR (Fig. 2a and b).

The lack of increased EGFR in the immunoprecipitate of IGF-1R in the resistant line could be due to the technical reasons such as the better precipitation capability of the EGFR antibody. Analysis of the supernatant after the immunoprecipitation by anti-EGFR revealed that almost 100 % of the protein had been removed from the lysate (Fig. 2c); by contrast about 33 % of the IGFR protein was left behind in the supernatant after immunoprecipitation (Fig. 2d). In addition, in the IGF1R immunoprecipitate, if there is a 1:1 stoichiometry, only 1/4^th^ of the EGFR molecules would be precipitated which could be undetectable. Taken together, these results suggest that cetuximab may activate EGFR to heterodimerize with IGF-1R and this interaction increases over time in resistant cells.

**Fig. 3:**
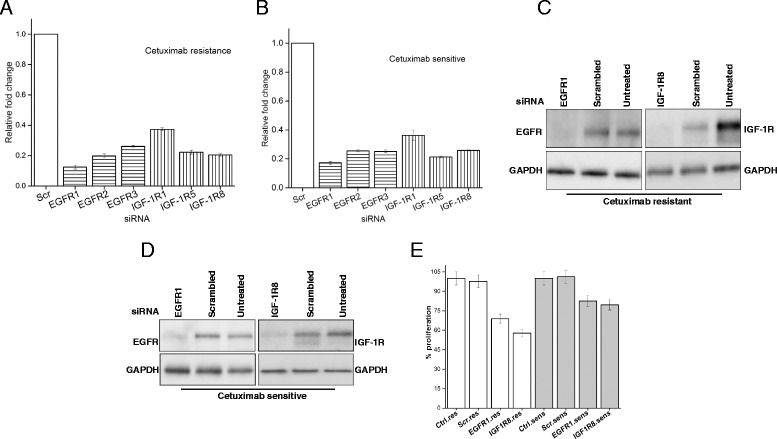
Knockdown of IGF-1R reduces the proliferation of resistant cells. **a** Relative expression of EGFR and IGF1R in resistant cells subjected to knockdown with three independent siRNAs targeting EGFR or IGF1R respectively after 60 h. Scrambled control was set to 1. **b** as in A but for the sensitive cell line. **c** Representative western blot indicating reduced levels of EGFR (left panel) or IGF1R (right panel) after knockdown with siRNA #1 and #8 respectively in resistant cells. GAPDH was used as a loading control protein was quantified at 60 h. **d** As in C but for the sensitive cell line. **e** Proliferation of resistant and sensitive cell lines in untreated, scrambled and specific knockdown conditions, measured using the CCK-8 assay, measured at 72 h. IGF1R has a greater effect on proliferation in resistant as compared to sensitive cells

We next determined if there was a functional consequence of increased IGF1R in the UMSCC-1 cetuximab resistant line. We performed siRNA-mediated knockdown of EGFR and IGF1R in both resistant and cell lines with 3 independent siRNAs and normalized the knockdown levels with four housekeeping genes (Additional file 1: Table S1). Efficient knockdown of up to 90 % was observed at the transcript level in both lines (Fig. 3a). Multiple siRNAs were tested to minimize off-target effects (Fig. 3a). Both EGFR and IGF1R levels were also reduced at the protein level (Fig. 3c and d) at 72 h post transfection of siRNA. Utilizing a cell viability assay we assessed the effect of the knockdown on cell proliferation after 72 h of depletion of the EGFR and IGF1R protein. Compared to the untreated and non-targeting control, under reduced levels of EGFR and IGF1R, there were reduced cell numbers in the resistant cell line of up to 67.98 and 57.8 % respectively (Fig. 3e). In contrast, at this time point the cell numbers in the sensitive were not significantly changed (Fig. 3e). These observations suggest that reducing levels of IGF1R can retard the growth rate of resistant cells suggesting a functional role.

**Fig. 4:**
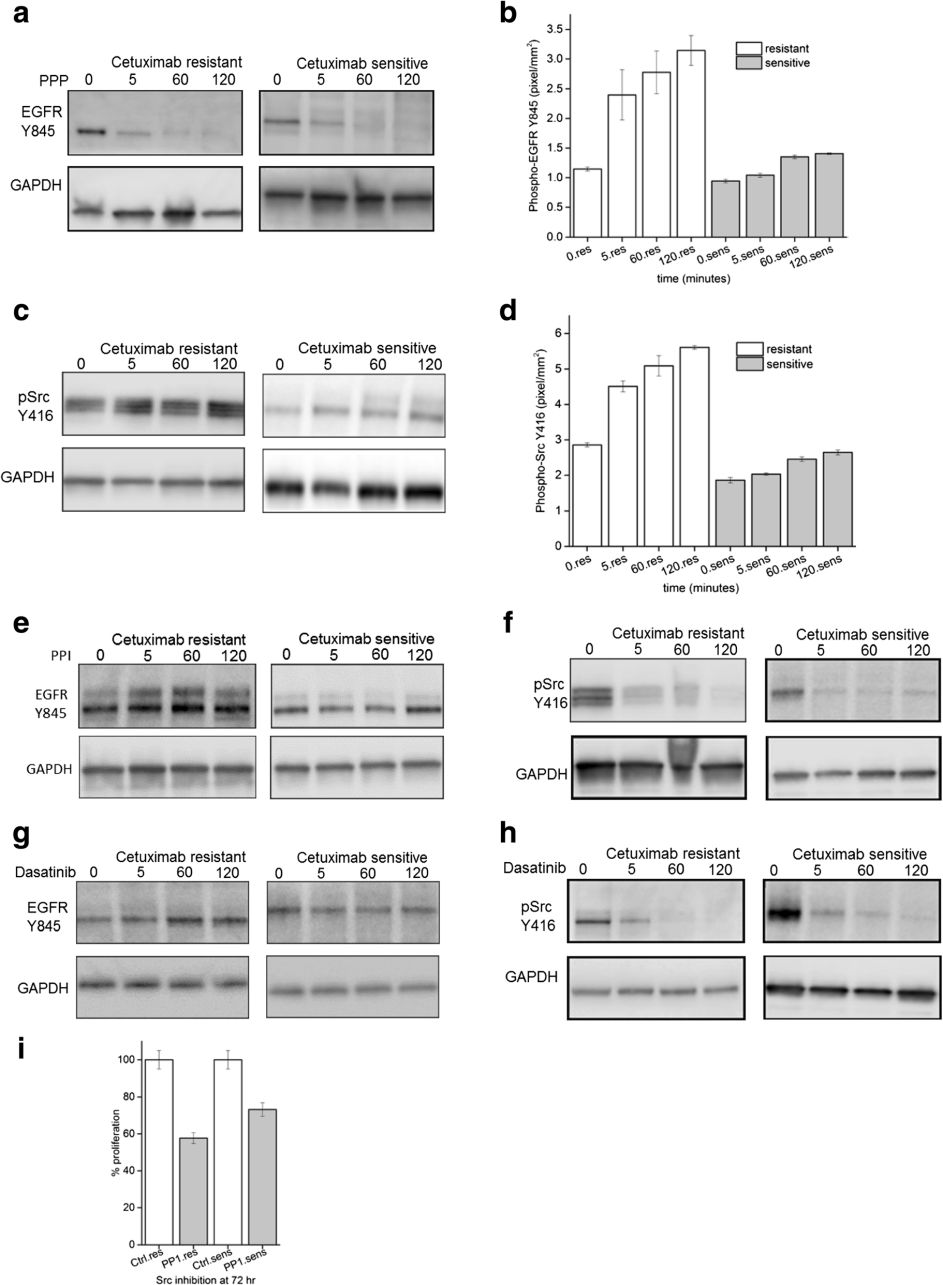
Phosphorylation of EGFR Y845 is not inhibited with Src inhibitors PP1 and Dasatinib. **a** EGFR Y845 levels show increased levels in response to cetuximab with time in resistant cells (*left panel*) and constant levels in sensitive cells (*right panel*). **b** Quantitation of EGFR Y845 normalized to GAPDH from four independent experiments after increasing times of exposure to10 μM of PP1. **c** phospho-Src Y416 show increased levels in response to cetuximab with time in resistant cells (*left panel*) and constant levels in sensitive cells (*right panel*). **d** Quantitation of p-Src Y416 normalized to GAPDH from four independent experiments after increasing times of exposure to cetuximab. **e** Representative western blot of EGFR Y845 show constant levels in response to PP1 in both resistant cells (*left panel*) and sensitive cells (*right panel*) in presence of cetuximab. **f** Representative western blot of p-Src 416 show reducing levels in response to PP1 in both resistant cells (*left panel*) and sensitive cells (*right panel*) with increasing time. **g** As in E above but after exposure to Dasatinib. **h** As in E above but after exposure to Dasatinib. **i** Proliferation of resistant and sensitive cell lines after exposure to PP1 for 72 h, measured using the CCK-8 assay

We next examined if heterodimerization of EGFR and IGF-1R caused any change in EGFR tyrosine phosphorylation states. Autophosphorylation sites Y992, Y1045 and Y1148 on EGFR revealed constitutive basal level of phosphorylation compared to untreated cells in the presence of cetuximab. However, Y1068 showed higher levels of phosphorylation in both resistant and sensitive cells compared to untreated cells suggesting an engagement of RAS signaling pathway (Additional file 1: Figure S3). While these autophosphorylation signals in the presence of cetuximab was intriguing in the presence of cetuximab, we focused on Tyrosine 845 in EGFR, which is present in the activation segment of the kinase domain of EGFR as it has been implicated in cell proliferation and migration in several cancers [24]. To address this, we probed for EGFR Y845 as a function of time and cetuximab treatment up to 2 h. There was an increase in Y845 phosphorylation in resistant cells relative to the untreated condition while levels remained relatively constant in sensitive cells (Fig. 4a). Y845 phosphorylation increased by 3-fold in 2 h (Fig. 4b).

The tyrosine kinase Src can phosphorylate EGFR at Y845 [25, 26]. Prior to Src activity on targets, autophosphorylation of its own catalytic site at position Y416 is required. Therefore we monitored whether Src-Y416 was also altered in its phosphorylation status in a similar manner to EGFR. We found the level of pSrc-Y416 was 1.60 fold higher in the resistant compared to sensitive cells (Fig. 4c and d). Furthermore, addition of cetuximab increased the levels of pSrc-Y416 about 2-fold in both resistant and sensitive cells at 120 min.

We next explored whether there was a relationship between the increase in p-SrcY416 and p-EGFR-Y845 without altering endogenous levels and hence used Src inhibitors in the presence of cetuximab. PP1with an IC50 of 170 nM is a potent selective dual site inhibitor of Src non-receptor tyrosine kinase family members which acts as a competitive inhibitor of ATP [27]. An inhibitor concentration of 10 μM previously reported to reduce the Src activity and level of EGFR Y845 [28] was used up to 120 min in both resistant and sensitive cells. Analyses of immunoblots from sensitive and resistant cells treated with PP1 inhibitor probed with Src family kinase-specific pY416 antibody revealed clearly that Src activity was diminished in both sensitive and resistant cells compared to untreated cells (Fig. 4f). Surprisingly, the levels of EGFR-Y845 did not reduce greatly in the presence of PP1and cetuximab, especially in the resistant cells (Fig. 4e). However, resistant and sensitive cells exposed to PP1 inhibitor showed reduced proliferation of approximately 60 and 76 % respectively (Fig. 4i) compared to untreated cells. To confirm this reduced effect of Src inhibition, we used Dasatinib, an alternative Src inhibitor with an IC50 of 0.8 nM. Similar to the PP1 results, in the presence of Dasatinib, pSrc-Y416 levels were reduced in both resistant and sensitive cells (Fig. 4h) while Y845 levels remained unchanged (Fig. 4g) compared to untreated cells. Quantification of Y845 signals from immunoblots represented in Fig. 4e, G further indicated that combined effect of PP1, dasatinib and cetuximab did not significantly diminish the phosphorylation state of EGFR Y845 (Additional file 1: Figure S2A, B). Taken together, the immediate early effects of cetuximab treatment suggested that EGFR Y845 levels increased despite the reduced activity at p-SrcY416. Furthermore, we tested if transactivation of EGFR Y845 could be mediated by IGF-1R as there was increased hetero-dimerization between EGFR and IGF-1R as observed in Fig. 2b. Indeed, siRNA mediated knock down of IGF-1R reduced Y845 phosphorylation state to almost undetectable levels (Additional file 1: Figure S1). This result further validated the co-immunoprecipitation of IGF-1R and EGFR (Fig. 2a, b) and IGF-1R’s plausible role in phosphorylating EGFR Y845 phosphorylation in the presence of cetuximab.

**Fig. 5:**
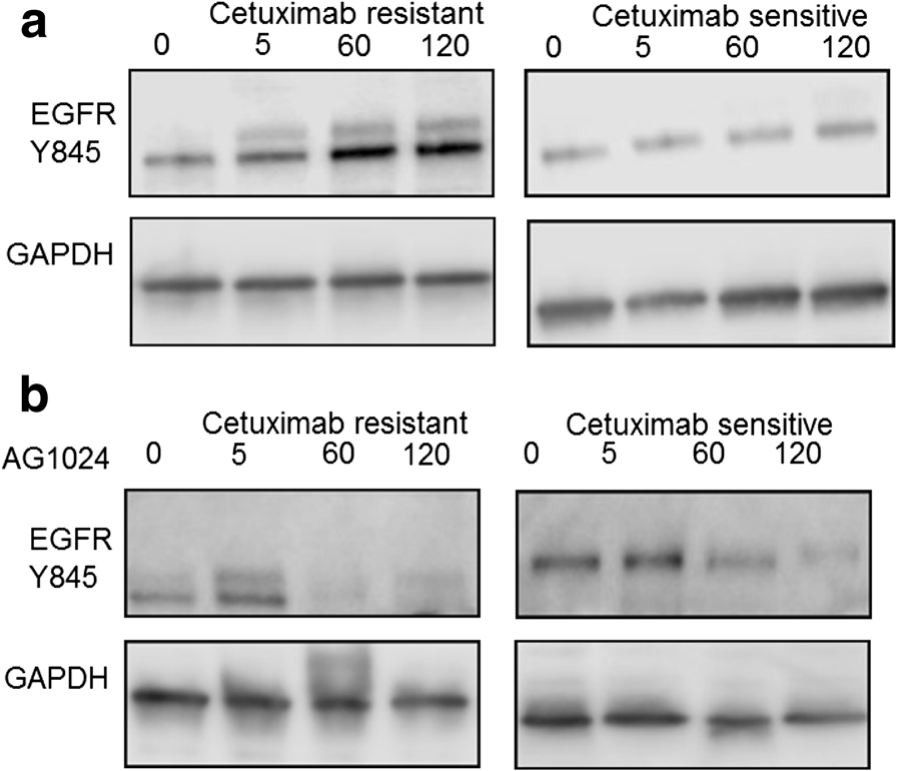
Phosphorylation of EGFR Y845 is reduced with IGF-1R specific inhibitor picropodophyllin (PPP). **a** Representative western blot of EGFR Y845 show decreased levels in response to 0.5 μM concentration PPP with time in both resistant cells (*left panel*) and in sensitive cells (*right panel*) The inhibitory effect of PPP is more rapid in resistant as compared to sensitive cells. **b** similar treatment times as in (**a**), but monitored effect using 10 μM AG1024, another inhibitor of IGF-1R. Note that AG1024 is less inhibitory to IGF1R than PPP

Like EGFR, IGF-1R is involved in normal cellular growth as well as transformation and progression of malignancy in various cancers [29]. PPP with an IC50 of 1 nM is a specific inhibitor of IGF-1R. To determine if inhibition of kinase activity of IGF-1R had an influence on EGFR Y845 due to their hetero-dimerization, resistant and sensitive cells were exposed to picropodophyllin (PPP) at 0.5 μM for 120 min. Interestingly, PPP completely reduced Y845 phosphorylation at 120 min in both resistant and sensitive cells compared to untreated cells, (Fig. 5a). To confirm these results, we tested another inhibitor of IGF-1R, AG1024 which has an IC50 of 0.4 μM. AG1024 effects were more gradual than that of PPP showing reduced levels of Y845 only at later time points in the resistant line, (Fig. 5b). This data suggests that in the resistant line, where there is more heterodimerization of EGFR-IGF1R leading to greater Y845 levels, the less potent inhibitor AG1024 can act rapidly. In the sensitive cell line these heterodimers are less frequent; hence the potent PPP inhibitor can act to remove the phosphorylation at Y845 rapidly. A combined treatment of resistant and sensitive cells with PPP and cetuximab showed 39 and 54 % reduced proliferation while PPP showed a reduction of 51 and 41 % (Additional file 1: Figure S4A) while a similar trend of reduced proliferation was also observed with another IGF-1R inhibitor,AG1024 (Additional file 1: Figure S4B). This result suggested that PPP and AG1024 can act together with cetuximab to impede the cell growth of these cell lines.

## Discussion

Systematic preclinical investigation of anti-EGFR signaling agents in human tumor cells and animal model systems has proven valuable in the logical design of clinical trials testing EGFR inhibitors [9]. As with virtually all anti-cancer drugs, chronic exposure to EGFR inhibitors eventually induces molecular changes that confer varying degrees of drug resistance. For the EGFR system, one mechanism of acquired resistance involves upregulation of collateral receptor tyrosine kinases (RTKs) that provide escape from the selection pressure of EGFR blockade [30–32]. For example, interactions between the EGFR, IGF-1R and VEGFR signaling pathways are well established and target inhibition within one system commonly impacts molecular signaling (and ultimately tumor response) via compensatory response from companion RTK pathways [10–12]. In the current study, we identify a specific molecular interaction between EGFR and IGF-1R that may play a role in resistance to cetuximab, the leading anti-EGFR inhibitory agent in current clinical use for cancer therapy. This may stimulate opportunities to further refine the response and resistance profile for cetuximab therapy in the future.

Signaling from the EGFR is affected by several parameters. For example the number of EGFR on the cell surface can affect the extent of signaling [33]. The ligand binding site of EGFR can have high or low affinity for EGF giving rise to two distinct population of receptors [34]. The outcome of EGFR signaling can vary with whether low or high affinity receptors are activated [35, 36]. EGFR can exist as monomers or preformed dimers on the cell surface [37]. Binding of EGF ligand promotes dimerization of the EGFR [38]. Since HNSCC cells overexpress EGFR, the number of dimers is likely to be increased. Binding of cetuximab leads to disruption of EGFR dimers in both resistant and sensitive HNSCC. The resultant effect is increased monomers of EGFR on the cell surface. Since IGF-1R levels are increased in resistant cells (Fig. 1b and c), this increases the probability of engagement of EGFR with IGF-1R causing heterodimerization (Fig. 2b). Preliminary observations using high resolution imaging suggests that there is increased clustering of EGFR and IGF-1R in the presence of cetuximab (unpublished results). This EGFR-IGF-1R interaction could provide an alternative signaling platform for maintenance of the resistant phenotype.

**Fig. 6:**
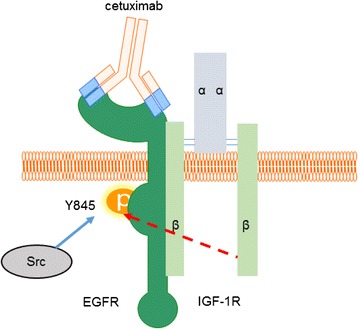
Model of cetuximab induced EGFR and IGF-1R interaction. Binding of anti-EGFR antibody cetuximab displaces the EGF ligand which results in disruption of EGFR homodimers. Cetuximab bound monomer of EGFR engages IGF-1R to form a heterodimer. The ligand independent activation of EGFR Y845 is not reduced in the presence of Src inhibitor PP1 (*dotted line*) but is reduced by IGF-1R inhibitor PPP. The model suggests that intrinsic kinase activity of IGF-1R has the potential to phosphorylate EGFR Y845 due to the heterodimerization of these receptors. This heterodimerization and hence sensitivity to IGF1R mediated phosphorylation is more pronounced in resistant than sensitive cells

EGFR-IGF-1R heterodimerization has been reported in drug resistant cancers, for example in non-small cell lung cancer (NSCLC), heterodimerization of EGFR and IGF-1R was increased upon exposure to the EGFR tyrosine kinase inhibitor, erlotinib [39], but the consequence of dimerization has remained poorly understood. In our study, we found that EGFR-IGF-1R interaction has a functional consequence of phosphorylation of EGFR Y845. The analysis of EGFR Y845 phosphorylation revealed it to be constitutively phosphorylated in both sensitive and resistant cells, albeit, the levels of EGFRY845 phosphorylation increased with time in the resistant cells (Fig. 4a). This site is a read out for EGFR activation and downstream signaling output [40]. The non-receptor tyrosine kinase Src is thought to be involved in EGFR Y845 phosphorylation [25, 26]. Furthermore, Src family kinases has been suggested to increase the EGFR Y845 levels in cetuximab resistant clones derived from lung NCI-H226 cells [41] and also implicated in erlotinib resistance in head and neck cancer [42]. However, abrogation of pSrc-Y416 phosphorylation by Src specific inhibitors PP1 and dasatinib did not decrease EGFR Y845 levels (Fig. 4e, g). In addition, siRNA knockdown also suggested reduced EGFR Y845 levels (Additional file 1: Figure S1). This result would suggest that heterocomplex of EGFR-IGF-1R in the presence of cetuximab may have adopted a conformation which prevents Src to access this Y845 located in the activation loop of EGFR (Fig. 6). Intriguingly, we observed the loss of EGFR Y845 phosphorylation upon treatment with IGF-1R inhibitor, PPP which suggested that heterodimerization is important for conferring this phosphorylation. Recent reports indicate that PPP has the ability to inhibit the phosphorylation status of EGFR in combination with erlotinib or alone in head and neck cancer [43].

Sharpening our understanding of resistance mechanisms to molecular targeting agents like cetuximab may illuminate opportunities to refine treatment strategies that increase tumor response and control rates. Our results suggest that a combinatorial approach that inhibits both EGFR and IGF1R signaling may offer a worthy strategy to enhance therapeutic outcome and combat acquired resistance to cetuximab in head and neck cancer. We are pursuing preclinical studies in tissue culture and animal model systems to further explore the potential value of this approach.

## Conclusions

Based on our observations, EGFR-IGF-1R interaction has a functional consequence of phosphorylation of EGFR Y845 in cetuximab resistant HNSCC cells. Targeting of EGFR and IGF-1R in a spatio-temporal manner could be a promising therapeutic strategy in head and neck cancer.

## Additional file

Additional file 1: Table S1. List of sequences for siRNA and housekeeping genes used in this study. **Figure S1.** Knockdown of IGF-1R diminishes the phosphorylation state of EGFR Y845. Representative western blot indicating reduced levels of EGFR Y845 in both cetuximab resistant and sensitive cells. GAPDH was used as a loading control protein was quantified at 60 h. **Figure S2.** Quantitation of phosphor-EGFR Y845 normalized to GAPDH from four independent experiments after increasing times of exposure to combined inhibition with PP1 and cetuximab. A) Addition of PP1 or B) dasatinib with cetuximab shows increased Y845 levels relatively in resistant cells treated while in sensitive cells, there is marginal increase suggesting lack of inhibition when Src inhibitors are combined with cetuximab. **Figure S3.** Effect of cetuximab on auto-phosphorylation sites of EGFR. Constitutive basal levels of autophosphorylation was detected for Y992, Y1045 and Y1148 in the presence of cetuximab in both resistant and sensitive cells while for Y1068 there was higher signal intensity compared to untreated cells suggesting immediate activation of the RAS signaling cascade in the presence of cetuximab. **Figure S4.** Combined inhibition of EGFR and IGF-1R leads to reduced proliferation compared to IGF-1R alone. A) Combination of 0.5 μM PPP, an IGF-1R inhibitor with 66.6 nM cetuximab leads to reduced cell proliferation when compared to single inhibitor treatment of PPP alone. B) A similar trend was also observed with another IGF-1R inhibitor, AG1024. All experiments were performed in quadruplicates thrice independently. (DOCX 441 kb)

## Abbreviations

AG1024: Tyrphostin
BCA: Bicinchoninic acid
CCK-8: Cell counting kit
EGFR: Epidermal growth factor receptor
GAPDH: Glyceraldehyde phosphate dehydrogenase
GPCR: G-protein coupled receptor
HNSCC: Head and neck squamous cell carcinoma
HPRT1: Hypoxanthine phosphoribosyl transferase
HPV: Human papillomavirus
IGF: Insulin growth factor
IGF-1R: Insulin like growth factor 1 like receptor
mAbs: Monoclonal antibodies
NSCLC: Non-small cell lung cancer
PP1: 1-(1,1-dimethylethyl)-3-(4-methylphenyl)-1H-pyrazolo[3,4-d]pyrimidin-4-amine
PPP: Picropodophyllin
TCGA: The cancer genome atlas
TKI: Tyrosine kinase inhibitor
UMSCC: University of Michigan squamous cell carcinoma
VEGFR: Vascular endothelial growth factor receptor

## References

[1] Ozanne B, Richards CS, Hendler F, Burns D, Gusterson B (1986). Over-expression of the EGF receptor is a hallmark of squamous cell carcinomas. J Pathol.

[2] Leemans CR, Braakhuis BJM, Brakenhoff RH (2011). The molecular biology of head and neck cancer. Nat Rev Cancer.

[3] The Cancer Genome Atlas N (2015). Comprehensive genomic characterization of head and neck squamous cell carcinomas. Nature.

[4] Chung CH (2004). Molecular classification of head and neck squamous cell carcinomas using patterns of gene expression. Cancer Cell.

[5] Arteaga Carlos L, Engelman Jeffrey A (2014). ERBB receptors: from oncogene discovery to basic science to mechanism-based cancer therapeutics. Cancer Cell.

[6] Bonner JA, Harari PM, Giralt J, Azarnia N, Shin DM, Cohen RB, Jones CU, Sur R, Raben D, Jassem J (2006). Radiotherapy plus cetuximab for squamous-cell carcinoma of the head and neck. N Engl J Med.

[7] Kies MS (2010). Induction chemotherapy and cetuximab for locally advanced squamous cell carcinoma of the head and neck: results from a phase II prospective trial. J Clin Oncol.

[8] Pietrantonio F, Cremolini C, Petrelli F, Di Bartolomeo M, Loupakis F, Maggi C, Antoniotti C, de Braud F, Falcone A, Iacovelli R (2015). First-line anti-EGFR monoclonal antibodies in pan<em>RAS</em> wild-type metastatic colorectal cancer: a systematic review and meta-analysis. Crit Rev Oncol Hematol.

[9] Harari PM, Huang S-M (2004). Combining EGFR inhibitors with radiation or chemotherapy: will preclinical studies predict clinical results?. Int J Radiat Oncol Biol Phys.

[10] Li J, Huang S, Armstrong EA, Fowler JF, Harari PM (2005). Angiogenesis and radiation response modulation after vascular endothelial growth factor receptor-2 (VEGFR2) blockade. Int J Radiat Oncol Biol Phys.

[11] Tabernero J (2007). The role of VEGF and EGFR inhibition: implications for combining anti–VEGF and anti–EGFR agents. Mol Cancer Res.

[12] Allen GW, Saba C, Armstrong EA, Huang S-M, Benavente S, Ludwig DL, Hicklin DJ, Harari PM (2007). Insulin-like growth factor-I receptor signaling blockade combined with radiation. Cancer Res.

[13] Wheeler DL, Huang S, Kruser TJ, Nechrebecki MM, Armstrong EA, Benavente S, Gondi V, Hsu K-T, Harari PM (2008). Mechanisms of acquired resistance to cetuximab: role of Her (ErbB) family members. Oncogene.

[14] Logue JS, Morrison DK (2012). Complexity in the signaling network: insights from the use of targeted inhibitors in cancer therapy. Genes Dev.

[15] Niederst MJ, Engelman JA. Bypass mechanisms of resistance to receptor tyrosine kinase inhibition in lung cancer. Sci Signal. 2013; 6(294): doi:10.1126/scisignal.2004652. 10.1126/scisignal.2004652 PMC3876281 24065147

[16] Bhola NE, Thomas SM, Freilino M, Joyce S, Sahu A, Maxwell J, Argiris A, Seethala R, Grandis JR (2011). Targeting GPCR-mediated p70S6K activity May improve head and neck cancer response to cetuximab. Clin Cancer Res.

[17] Pan S, Zhang H, Rush J, Eng J, Zhang N, Patterson D, Comb MJ, Aebersold R (2005). High throughput proteome screening for biomarker detection. Mol Cell Proteomics.

[18] Pollak M (2008). Insulin and insulin-like growth factor signalling in neoplasia. Nat Rev Cancer.

[19] Dale OT, Aleksic T, Shah KA, Han C, Mehanna H, Rapozo DCM, Sheard JDH, Goodyear P, Upile NS, Robinson M (2015). IGF-1R expression is associated with HPV-negative status and adverse survival in head and neck squamous cell cancer. Carcinogenesis.

[20] Klusmann J-H, Godinho FJ, Heitmann K, Maroz A, Lee Koch M, Reinhardt D, Orkin SH, Li Z (2010). Developmental stage-specific interplay of GATA1 and IGF signaling in fetal megakaryopoiesis and leukemogenesis. Genes Dev.

[21] Baker J, Liu J-P, Robertson EJ, Efstratiadis A (1993). Role of insulin-like growth factors in embryonic and postnatal growth. Cell.

[22] Jameson MJ, Beckler AD, Taniguchi LE, Allak A, VanWagner LB, Lee NG, Thomsen WC, Hubbard MA, Thomas CY (2011). Activation of the insulin-like growth factor-1 receptor induces resistance to epidermal growth factor receptor antagonism in head and neck squamous carcinoma cells. Mol Cancer Ther.

[23] Benavente S, Huang S, Armstrong EA, Chi A, Hsu K-T, Wheeler DL, Harari PM (2009). Establishment and characterization of a model of acquired resistance to epidermal growth factor receptor targeting agents in human cancer cells. Clin Cancer Res.

[24] Mueller KL, Powell K, Madden JM, Eblen ST, Boerner JL (2012). EGFR tyrosine 845 phosphorylation-dependent proliferation and transformation of breast cancer cells require activation of p38 MAPK. Transl Oncol.

[25] Sato KI, Sato A, Aoto M, Fukami Y (1995). c-SRC phosphorylates epidermal growth factor receptor on tyrosine 845. Biochem Biophys Res Commun.

[26] Biscardi JS, Maa M-C, Tice DA, Cox ME, Leu T-H, Parsons SJ (1999). c-Src-mediated phosphorylation of the epidermal growth factor receptor on Tyr845 and Tyr1101 is associated with modulation of receptor function. J Biol Chem.

[27] Hanke JH, Gardner JP, Dow RL, Changelian PS, Brissette WH, Weringer EJ, Pollok BA, Connelly PA (1996). Discovery of a novel, potent, and Src family-selective tyrosine kinase inhibitor: study of Lck - and FynT- dependent T cell activation. J Biol Chem.

[28] Koppikar P, Choi S-H, Egloff AM, Cai Q, Suzuki S, Freilino M, Nozawa H, Thomas SM, Gooding WE, Siegfried JM (2008). Combined inhibition of c-Src and epidermal growth factor receptor abrogates growth and invasion of head and neck squamous cell carcinoma. Clin Cancer Res.

[29] Girnita A, Girnita L, Prete F, Bartolazzi A, Larsson O, Axelson M (2004). Cyclolignans as inhibitors of the insulin-like growth factor-1 receptor and malignant cell growth. Cancer Res.

[30] Azuma K, Kawahara A, Sonoda K, Nakashima K, Tashiro K, Watari K, Izumi H, Kage M, Kuwano M, Ono M (2014). FGFR1 activation is an escape mechanism in human lung cancer cells resistant to afatinib, a pan-EGFR family kinase inhibitor. Oncotarget.

[31] Chakravarti A, Loeffler JS, Dyson NJ (2002). Insulin-like growth factor receptor I mediates resistance to anti-epidermal growth factor receptor therapy in primary human glioblastoma cells through continued activation of phosphoinositide 3-kinase signaling. Cancer Res.

[32] Sawano A, Takayama S, Matsuda M, Miyawaki A (2002). Lateral propagation of EGF signaling after local stimulation is dependent on receptor density. Dev Cell.

[33] Shoyab M, De Larco JE, Todaro GJ (1979). Biologically active phorbol esters specifically alter affinity of epidermal growth factor membrane receptors. Nature.

[34] Krall JA, Beyer EM, MacBeath G (2011). High- and low-affinity epidermal growth factor receptor-ligand interactions activate distinct signaling pathways. PLoS One.

[35] Defize LH, Boonstra J, Meisenhelder J, Kruijer W, Tertoolen LG, Tilly BC, Hunter T, van Bergen en Henegouwen PM, Moolenaar WH, de Laat SW (1989). Signal transduction by epidermal growth factor occurs through the subclass of high affinity receptors. J Cell Biol.

[36] Tao R-H, Maruyama IN (2008). All EGF(ErbB) receptors have preformed homo- and heterodimeric structures in living cells. J Cell Sci.

[37] Dawson JP, Berger MB, Lin C-C, Schlessinger J, Lemmon MA, Ferguson KM (2005). Epidermal growth factor receptor dimerization and activation require ligand-induced conformational changes in the dimer interface. Mol Cell Biol.

[38] Nahta R, Yuan LXH, Zhang B, Kobayashi R, Esteva FJ (2005). Insulin-like growth factor-I receptor/human epidermal growth factor receptor 2 heterodimerization contributes to trastuzumab resistance of breast cancer cells. Cancer Res.

[39] K-i S (2013). Cellular functions regulated by phosphorylation of EGFR on Tyr845. Int J Mol Sci.

[40] Wheeler DL, Iida M, Kruser TJ, Nechrebecki MM, Dunn EF, Armstrong EA, Huang S, Harari PM (2009). Epidermal growth factor receptor cooperates with Src family kinases in acquired resistance to cetuximab. Cancer Biol Ther.

[41] Stabile LP, He G, Lui VWY, Thomas SM, Henry C, Gubish CT, Joyce S, Quesnelle KM, Siegfried JM, Grandis JR (2013). c-Src activation mediates erlotinib resistance in head and neck cancer by stimulating c-Met. Clin Cancer Res.

[42] Clayburgh DR, Gross ND, Proby C, Koide J, Wong MH (2013). Effects of epidermal growth factor receptor and insulin-like growth factor 1 receptor inhibition on proliferation and intracellular signaling in cutaneous SCCHN: potential for dual inhibition as a therapeutic modality. Head Neck.

[43] Yin S-C, Guo W, Tao Z-Z (2013). Picropodophyllin inhibits tumor growth of human nasopharyngeal carcinoma in a mouse model. Biochem Biophys Res Commun.

