# Erratum to: Insulin-like growth factor 1 receptor mediated tyrosine 845 phosphorylation of epidermal growth factor receptor in the presence of monoclonal antibody cetuximab

**DOI:** 10.1186/s12885-016-2958-x

**Published:** 2016-12-01

**Authors:** Gopal Iyer, James Price, Shay Bourgeois, Eric Armstrong, Shyhmin Huang, Paul M. Harari

**Affiliations:** Department of Human Oncology, University of Wisconsin School of Medicine and Public Health, University of Wisconsin-Madison, Madison, 53792 USA

## Erratum

After the publication of article [1], it is noticed that the article title was incorrect. The correct title should be: “Insulin-like growth factor 1 receptor mediated tyrosine 845 phosphorylation of epidermal growth factor receptor in the presence of monoclonal antibody cetuximab”. Please note that this has been corrected in the above title. We apologise for any inconvenience caused.
