# Supplementary material for: Insulin growth factor 1 like receptor (IGF-1R)
Source: BMC Cancer. 2016 Oct 6;16:773. doi: 10.1186/s12885-016-2796-x (PMC5054590; doi:10.1186/s12885-016-2796-x)
Supplement: Additional file 1: Table S1. — List of sequences for siRNA and housekeeping genes used in this study. Figure S1. Knockdown of IGF-1R diminishes the phosphorylation state of EGFR Y845. Representative western blot indicating reduced levels of EGFR Y845 in both cetuximab resistant and sensitive cells. GAPDH was used as a loading control protein was quantified at 60 h. Figure S2. Quantitation of phosphor-EGFR Y845 normalized to GAPDH from four independent experiments after increasing times of exposure to combined inhibition with PP1 and cetuximab. A) Addition of PP1 or B) dasatinib with cetuximab shows increased Y845 levels relatively in resistant cells treated while in sensitive cells, there is marginal increase suggesting lack of inhibition when Src inhibitors are combined with cetuximab. Figure S3. Effect of cetuximab on auto-phosphorylation sites of EGFR. Constitutive basal levels of autophosphorylation was detected for Y992, Y1045 and Y1148 in the presence of cetuximab in both resistant and sensitive cells while for Y1068 there was higher signal intensity compared to untreated cells suggesting immediate activation of the RAS signaling cascade in the presence of cetuximab. Figure S4. Combined inhibition of EGFR and IGF-1R leads to reduced proliferation compared to IGF-1R alone. A) Combination of 0.5 μM PPP, an IGF-1R inhibitor with 66.6 nM cetuximab leads to reduced cell proliferation when compared to single inhibitor treatment of PPP alone. B) A similar trend was also observed with another IGF-1R inhibitor, AG1024. All experiments were performed in quadruplicates thrice independently. (DOCX 441 kb) [file 12885_2016_2796_MOESM1_ESM.docx]

| Gene | Forward | Reverse |
| --- | --- | --- |
| siEGFR1 | GGAAATTACCTATGTGCAGAGGAAT | ATTCCTCTGCACATAGGTAATTTCCAA |
| siEGFR2 | CCAGTATTGATCGGGAGAGCCGGAG | CTCCGGCTCTCCCGATCAATACTGGAC |
| siEGFR3 | GGCTGGTTATGTCCTCATTGCCCTC | GAGGGCAATGAGGACATAACCAGCCAC |
| siIGF-1R1 | GTGATAAATTACCAGTTTCAATCAC | GTGATTGAAACTGGTAATTTATCACAA |
| siIGF-1R5 | AAGGATATTGGGCTTTACAACCTGA | TCAGGTTGTAAAGCCCAATATCCTTGA |
| siIGF-1R8 | CATCATGGAATTGATGTGAGCATTA | TAATGCTCACATCAATTCCATGATGTG |
| β-Actin | CCTTGCACATGCCGGAG | ACAGAGCCTCGCCTTTG |
| β-Microglobulin | ACCTCCATGATGCTGCTTAC | GGACTGGTCTTTCTATCTCTTGT |
| Glyceraldehyde-3-Phosphate Dehydrogenase (GAPDH) | TGTAGTTGAGGTCAATGAAGGG | ACATCGCTCAGACACCATG |
| Hypoxanthine phosphoribosyltransferase 1 (HPRT1) | GCGATGTCAATAGGACTCCAG | TTGTTGTAGGATATGCCCTTGA |

**Additional file 1**

**Table S1:** Primer sequences for housekeeping genes and siRNAs

**
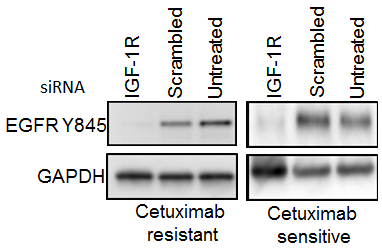
**

**Figure S1. Knockdown of IGF-1R diminishes the phosphorylation state of EGFR Y845.** Representative western blot indicating reduced levels of EGFR Y845 in both cetuximab resistant and sensitive cells. GAPDH was used as a loading control protein was quantified at 60 hours.

B

A


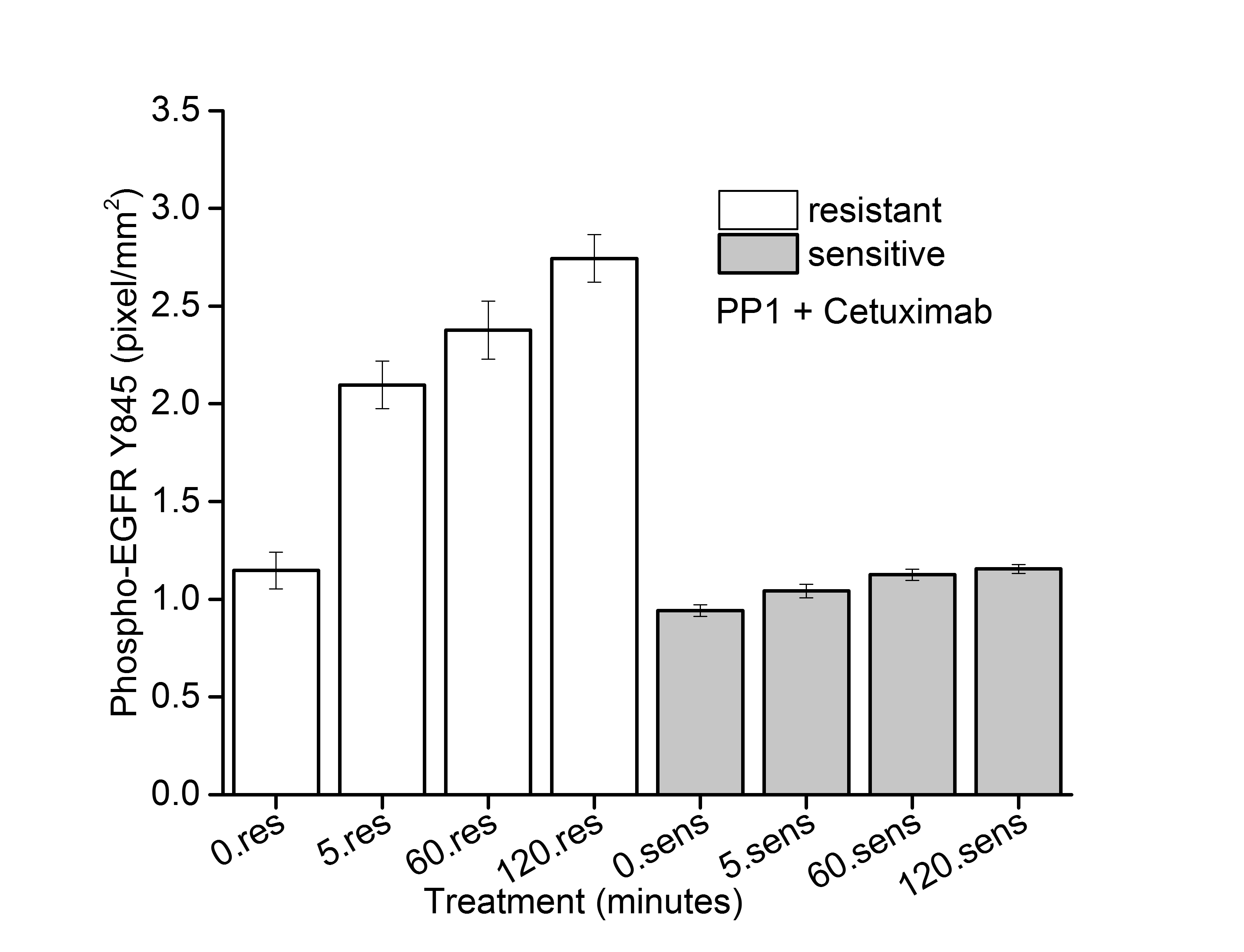

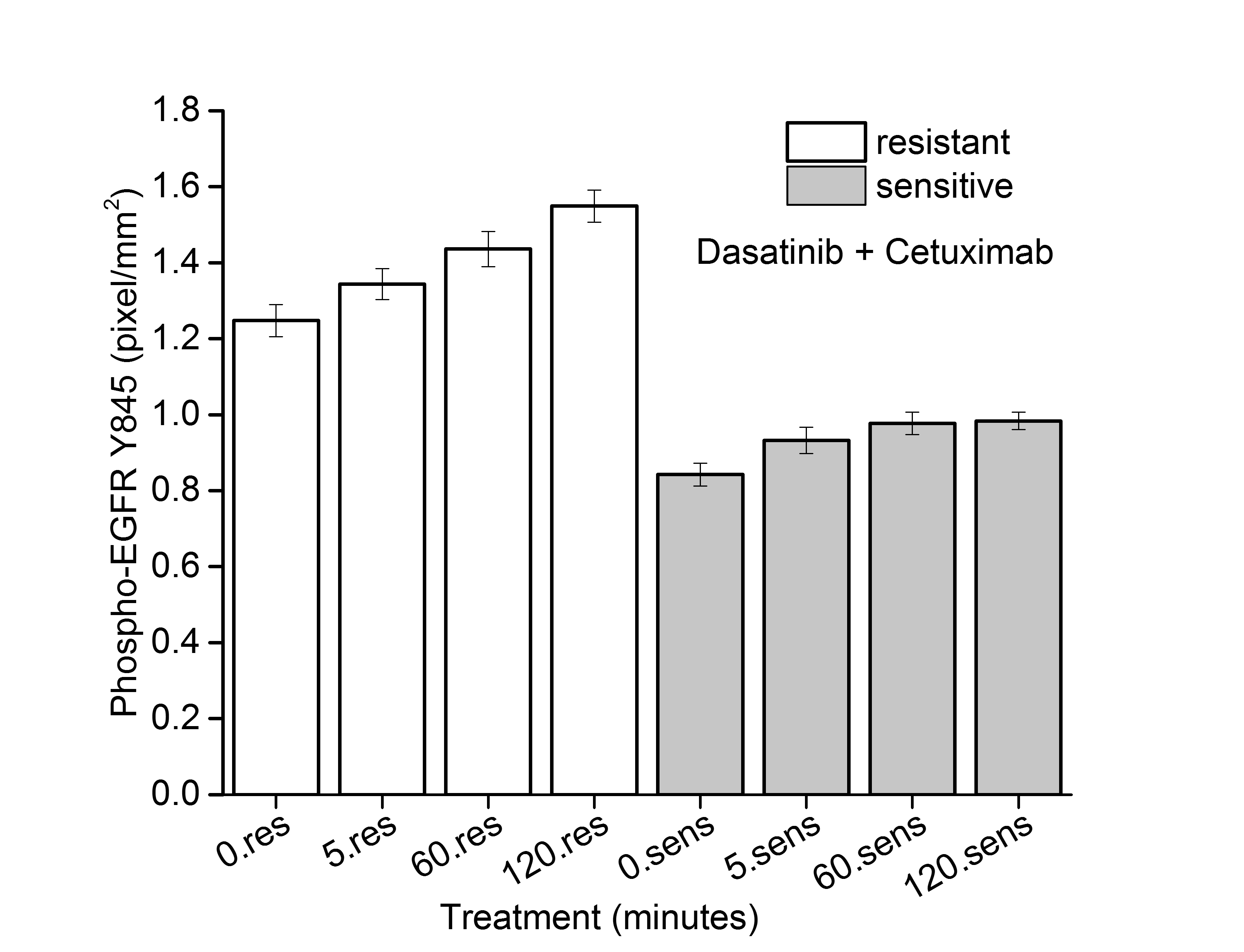


**Figure S2. Quantitation of phosphor-EGFR Y845 normalized to GAPDH from four independent experiments after increasing times of exposure to combined inhibition with PP1 and cetuximab. A)** Addition of PP1 or **B)** dasatinib with cetuximab shows increased Y845 levels relatively in resistant cells treated while in sensitive cells, there is marginal increase suggesting lack of inhibition when Src inhibitors are combined with cetuximab.


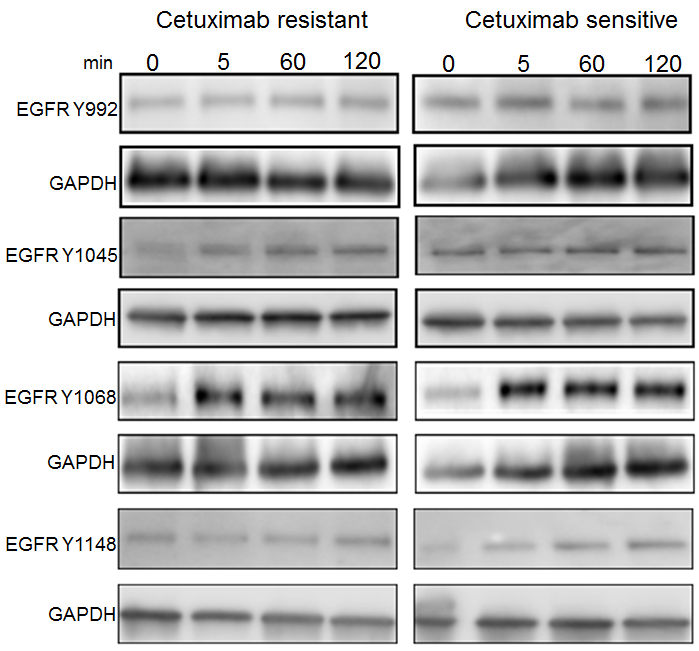
**Figure S3. Effect of cetuximab on auto-phosphorylation sites of EGFR***.* Constitutive basal levels of autophosphorylation was detected for Y992, Y1045 and Y1148 in the presence of cetuximab in both resistant and sensitive cells while for Y1068 there was higher signal intensity compared to untreated cells suggesting immediate activation of the RAS signaling cascade in the presence of cetuximab.

**
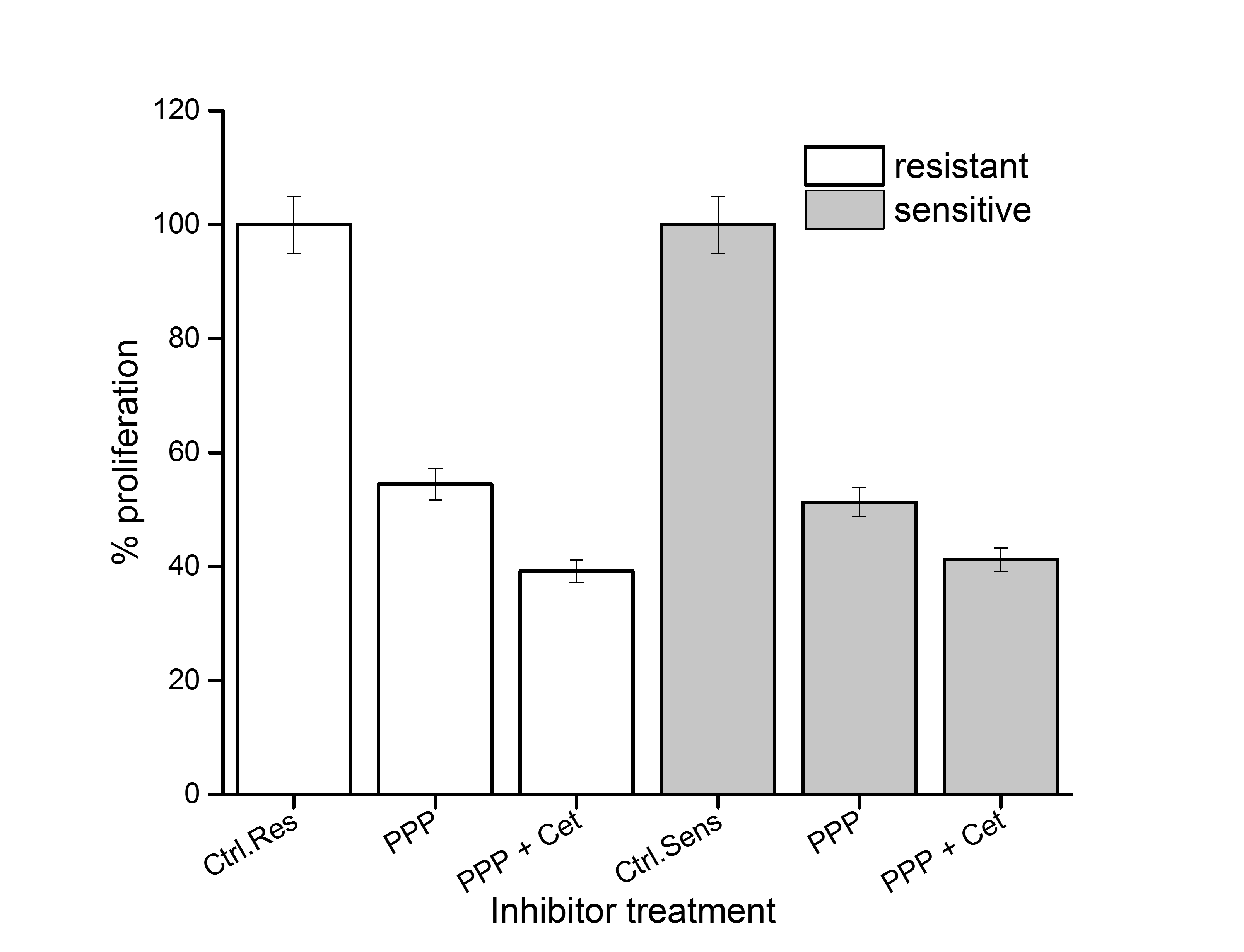

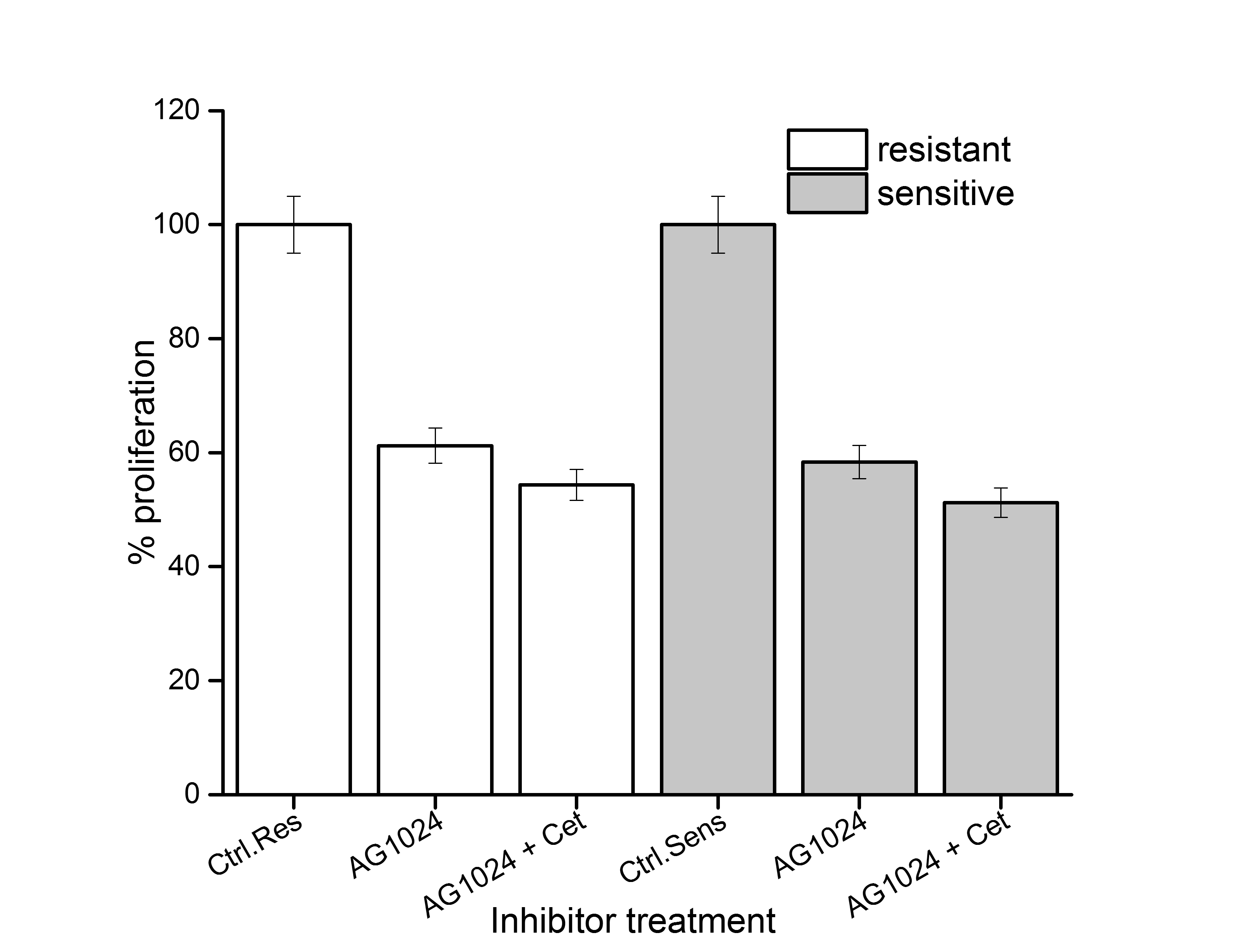
**

B

A

**Figure S4. Combined inhibition of EGFR and IGF-1R leads to reduced proliferation compared to IGF-1R alone. A)** Combination of 0.5 µM PPP, an IGF-1R inhibitor with 66.6 nM cetuximab leads to reduced cell proliferation when compared to single inhibitor treatment of PPP alone. **B)** A similar trend was also observed with another IGF-1R inhibitor, AG1024. All experiments were performed in quadruplicates thrice independently.
